# The outcomes and decision-making process for neck lymph nodes with indeterminate fine-needle aspiration cytology

**DOI:** 10.1371/journal.pone.0246437

**Published:** 2021-02-04

**Authors:** Ping-Chia Cheng, Chih-Ming Chang, Li-Jen Liao, Po-Wen Cheng, Wu-Chia Lo

**Affiliations:** 1 Department of Otolaryngology Head and Neck Surgery, Far Eastern Memorial Hospital, New Taipei City, Taiwan (R.O.C.); 2 Department of Biomedical Engineering, National Yang-Ming University, Taipei, Taiwan (R.O.C.); 3 Department of Electrical Engineering, Yuan Ze University, Taoyuan, Taiwan (R.O.C.); 4 Medical Engineering Office, Far Eastern Memorial Hospital, New Taipei City, Taiwan (R.O.C.); Brigham and Women’s Hospital, Harvard Medical School, UNITED STATES

## Abstract

**Objectives:**

This study aims to propose a cytological classification, to evaluate predictive factors of the final malignancy, and to suggest a proper management strategy for neck lymph nodes (LNs) with indeterminate cytology.

**Methods:**

Patients who had neck lymphadenopathy with indeterminate cytology between 2007 and 2017 were analyzed retrospectively in a tertiary medical center. Cytological classification was conducted according to the cytological descriptions. We examined the clinical characteristics according to the final diagnosis of the neck lymphadenopathy.

**Results:**

According to the final diagnoses, there were 142 malignant and 95 benign neck LNs among 237 patients. Multivariate analyses using a stepwise logistic regression model showed that cytological classification [p < 0.001, OR = 5.67 (3.48–9.23)], prior history of malignancy [p = 0.01, OR = 2.97 (1.26–6.99)], long axis [p = 0.01, OR = 3.06 (1.33–7.06)], short-to-long axis (S/L) ratio [p = 0.047, OR = 2.15 (1.01–4.57)] and internal echogenicity [p = 0.01, OR = 2.72 (1.26–5.86)] were independent predictors of malignancy.

**Conclusions:**

In patients who have neck LNs with indeterminate cytology, a cytological classification and four other predictors (prior history of malignancy, long axis ≥ 1.93 cm, S/L ratio ≥ 0.64 and heterogeneity of internal echogenicity) are statistically associated with the risk of malignancy and helpful in guiding further management.

## Introduction

Neck lymphadenopathy (LAP) is a common problem that causes patients to visit surgeons. The incidence rate of neck LAP is approximately 0.6% to 0.7% per year [1]. The common causes include reactive hyperplasia, tuberculous lymphadenitis, other granulomatous lymphadenitis, metastatic tumor and lymphoma [1–3]. For the evaluation of neck LAP, ultrasound (US) and US-guided fine-needle aspiration (US-FNA) are widely used due to their low cost, ease of operation and capability for simultaneous sampling. The sensitivity, specificity and accuracy rates of FNA for diagnosing malignant neck LAP range from 78% to 98% [2, 4–8], 67% to 100% [2, 4–8] and 82% to 97% [4, 6, 8], respectively. Despite the good performance of US-FNA, some cytological reports may not demonstrate a definite diagnosis even with adequate sampling. A previous study defined this cytological group as an indeterminate group [9]. They also classified the cytological reports of FNA into 4 groups: nondiagnostic (inadequate sample), benign, indeterminate, and malignant [9]. For neck lymph node (LN) aspirations, indeterminate cytology accounted for 8% to 10% of all FNA cytological reports [3, 9]. Bandoh et al. showed that the malignancy rate of indeterminate cytology was 79% (11 of 14) [9]. Tarantino et al. presented that the malignancy rate of atypical cytology was 77% (10 of 13) [3]. However, their case numbers were small.

Currently, no clear guidelines, such as the Bethesda system utilized for thyroid nodules, exist with regard to clinical decision-making when faced with a cervical LN with indeterminate FNA cytology. In addition, we all know that some characteristics, such as demographic and sonographic features, have been applied to assess the final malignancy risk of neck LNs [10, 11]. The purposes of this study are thus threefold: 1) to propose a cytological classification system for indeterminate cytology and to examine whether the new system is a practical method for determining the final malignancy, 2) to evaluate if demographic and sonographic features could help to predict the final malignancy in these patients, and 3) to suggest an effective management strategy when facing a neck LN with indeterminate FNA cytology.

## Materials and methods

### Ethical considerations

This study was approved by the institutional ethical review board of Far Eastern Memorial Hospital [IRB No. 107102-E]. The study did not influence the patients’ treatment or outcome. All data were retrospectively collected using a de-identified form between January 2020 and March 2020. We then analyzed the results from this anonymized data set. The final data set of the current study is within the S1 Table.

### Inclusion and exclusion criteria

We performed a retrospective study at a tertiary medical center. We followed the strengthening the reporting of observational studies in epidemiology (STROBE) statement in this study. Patients who received US and US-FNA due to neck LAP from October 2007 to September 2017 were reviewed. We divided the cytological reports into four classifications: nondiagnostic, benign, indeterminate, and malignant. The patients who had indeterminate cytological reports were included and subsequently analyzed. All patients either received core needle biopsy, excisional biopsy, neck dissection or follow-up for at least 1 year to obtain the final diagnosis. Patients who did not have histological reports or were lost to follow-up for over one year after the cytological reports were excluded.

### Clinical characteristics and outcomes assessment

The age, sex, side of LAP, prior history of malignancy and cytological reports were recorded from the medical charts. We assumed that the level of atypia was related to the malignancy rate. Thus, we further classified patients with indeterminate cytology into low-, moderate- and high-risk groups according to the cytological descriptions. The low-risk group included those with mild atypia, focal atypical cells, or tumor necrosis in the cytological report. The moderate-risk group includes those with atypia or some atypical cells in the description. The high-risk group included those with a statement of highly atypia, highly atypical cells or suspicion of malignancy in the report. These cytological descriptions were made by our cytopathologists after examining the cellularity, nuclear/cytoplasmic ratio, nuclear hyperchromatism, mitotic features, and nuclear outline [12]. We unbiasedly reviewed the formal reports and divided these indeterminate statements into 3 groups. The US findings of short and long axis, short-to-long axis (S/L) ratio, and other sonographic features, including boundary, internal echogenicity, echogenicity, calcifications, architecture, hilar echogenicity and vascular pattern of the neck LNs, were retrieved from a Marosis PACS system (Marotech Inc., Seoul, South Korea). We analyzed the demographic data and sonographic features according to the final diagnosis of the neck LAP.

### Statistical analysis

Statistical analysis was performed using STATA software, version 12.0 (Stata Corporation, College Station, TX). Statistical significance was defined as p < 0.05. Categorical variables were compared using the chi-squared or Fisher’s exact test, while continuous variables were compared using the two-sample t-test. Multivariate analyses were performed to identify the risk factors of a final malignancy by using a stepwise logistic regression model adjusted by age and sex. We defined the significance level as 0.05 for removal from the model. Odds ratios (ORs) with 95% confidence intervals (95% CIs) were reported.

## Results

There were 3393 patients who underwent US-FNA in our department from October 2007 to September 2017. Among them, 237 patients (7%, 273/3393) who had 237 LNs with indeterminate cytology were analyzed in the study (Table 1). The mean (SD) age of these patients was 50 (16) years, ranging from 12 to 88 years. The mean (SD) short and long axes of LAP were 1.19 (0.66) and 1.93 (1.10) cm, respectively. The mean (SD) S/L ratio was 0.64 (0.17). We further used the mean values of the above factors to dichotomize these patients.

**Table 1:** Demographic data of the patients who had neck LNs with indeterminate cytology.

Demographic data	N = 237
Age, mean (SD), yrs	50 (16)
Sex, No. (%)	
Female	81 (34%)
Male	156 (66%)
Side, No. (%)	
Right	108 (46%)
Left	102 (43%)
Bilateral	27 (11%)
Short axis, mean (SD), cm	1.19 (0.66)
Long axis, mean (SD), cm	1.93 (1.10)
S/L ratio, mean (SD)	0.64 (0.17)
Prior history of malignancy, No. (%)	92 (39%)
Cytological classification, No. (%)	
Low risk	72 (30%)
Moderate risk	62 (26%)
High risk	103 (44%)
**Final diagnoses**	
Malignant lymph nodes (N = 142, 60%)	
Oral cancer	33
Lymphoma	27
Thyroid cancer	26
Nasopharyngeal carcinoma	14
Unknown primary neck cancer	9
Hypopharyngeal cancer	8
Laryngeal cancer	6
Oropharyngeal cancer	5
Lung cancer	4
Breast cancer	3
Esophageal cancer	2
Parotid cancer	1
Conjunctival cancer	1
Bladder cancer	1
Cervical cancer	1
Colon cancer	1
Benign lymph nodes (N = 95, 40%)	
Reactive hyperplasia	83
Tuberculous lymphadenitis	6
Kikuchi disease	3
Toxoplasmic lymphadenitis	1
Other granulomatous lymphadenitis	2

Abbreviation: S/L, short-to-long axis; LNs, lymph nodes.

**Fig 1:**
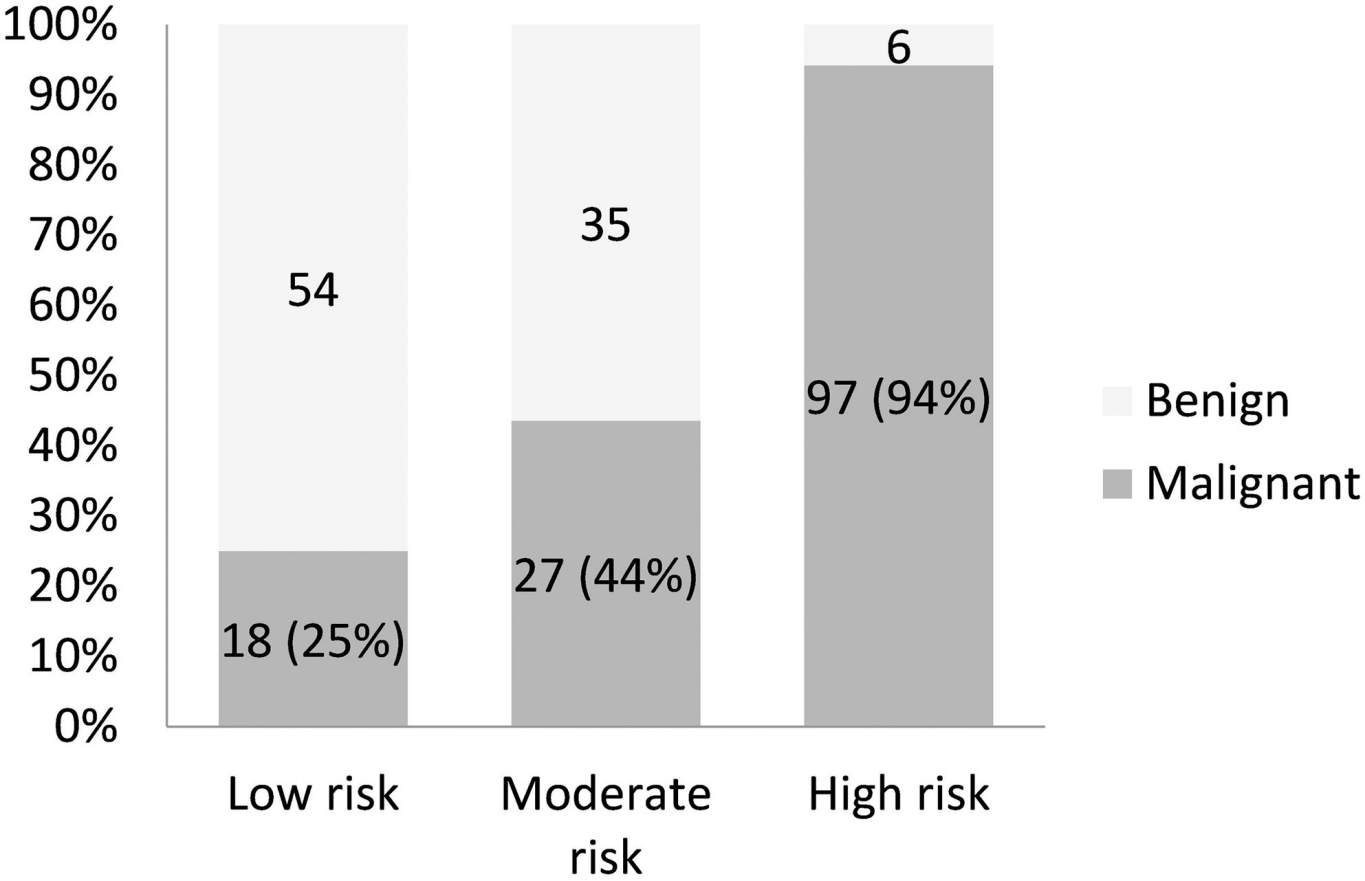
Final malignancy rates of indeterminate cytology among different cytological classifications.

According to the final diagnosis, there were 142 malignant and 95 benign LNs. The malignancy rate was 60% in these LNs. Among the malignant results, oral cancer, lymphoma and thyroid cancer were the most common diagnoses (Table 1). Among the indeterminate cytology results, the final malignancy rate was highest in the high-risk group [94% (97 of 103)], followed by the moderate-risk group [44% (27 of 62)] and low-risk group [25% (18 of 72)] (Fig 1).

The demographic data and sonographic features were compared between benign and malignant LNs according to the final diagnosis. There were significant differences in age (p = 0.001), sex (p < 0.001), short axis (p < 0.001), long axis (p < 0.001), S/L ratio (p = 0.002), prior history of malignancy (p < 0.001), cytological classification (p < 0.001), boundary (p < 0.001), internal echogenicity (p < 0.001), calcifications (p < 0.001), hilar echogenicity (p < 0.001) and vascular pattern (p = 0.03) between groups, but no significant differences in the side of LAP or other sonographic features including echogenicity and architecture were observed (Table 2).

**Table 2:** Comparison of demographic and sonographic features between patients with benign and malignant final diagnoses from neck LNs with indeterminate cytology.

	Final Diagnoses		
	Benign	Malignant	
Variables, No. (%)	N = 95 (40%)	N = 142 (60%)	P value
**Demographic data**			
Age			**0.001** ^a)^
<50 yrs	58 (51%)	55 (49%)	
≥50 yrs	37 (30%)	87 (70%)	
Sex			**< 0.001** ^a)^
Female	46 (57%)	35 (43%)	
Male	49 (31%)	107 (69%)	
Side			0.82
Right	41 (38%)	67 (62%)	
Left	43 (42%)	59 (58%)	
Bilateral	11 (41%)	16 (59%)	
Short axis (mean = 1.19 cm)			**< 0.001** ^a)^
<1.19 cm	68 (53%)	61 (47%)	
≥1.19 cm	27 (25%)	81 (75%)	
Long axis (mean = 1.93 cm)			**< 0.001** ^a)^
<1.93 cm	72 (50%)	73 (50%)	
≥1.93 cm	23 (25%)	69 (75%)	
S/L ratio (mean = 0.64)			**0.002** ^a)^
<0.64	57 (50%)	56 (50%)	
≥0.64	38 (31%)	86 (69%)	
Prior history of malignancy			**< 0.001** ^a)^
Absent	71 (49%)	74 (51%)	
Present	24 (26%)	68 (74%)	
Cytological classifications			**< 0.001** ^a)^
Low risk	54 (75%)	18 (25%)	
Moderate risk	35 (56%)	27 (44%)	
High risk	6 (6%)	97 (94%)	
**Sonographic feature**			
Boundary			**<0.001** ^a)^
Clear	79 (48%)	86 (52%)	
Vague	16 (22%)	56 (78%)	
Internal echogenicity			**<0.001** ^a)^
Homogenous	74 (54%)	63 (46%)	
Heterogeneous	21 (21%)	79 (79%)	
Echogenicity			0.21 ^b)^
Hyper	0 (0%)	4 (100%)	
Iso	3 (30%)	7 (70%)	
Hypo	92 (41%)	131 (59%)	
Calcification			**<0.001** ^a)^
Absent	89 (45%)	108 (55%)	
Present	6 (15%)	34 (85%)	
Architecture			0.41
Cystic	7 (32%)	15 (68%)	
Solid	88 (41%)	127 (59%)	
Hilar echogenicity			**<0.001** ^a)^
Absent	65 (33%)	131 (67%)	
Present	30 (73%)	11 (27%)	
Vascular pattern			**0.03** ^a)^
Hilar or avascular	79 (44%)	100 (56%)	
Other	16 (28%)	42 (72%)	

Abbreviation: S/L, short-to-long axis; LNs, lymph nodes.

^a)^Statistical significance, p < 0.05.

^b)^Calculated with Fisher’s exact test.

Multivariate analyses performed by using a stepwise logistic regression model adjusted for age and sex showed that cytological classification had the highest OR (5.67, 95% CI: 3.48–9.23), with a significant difference between groups (p < 0.001). The other independent risk factors for a final malignancy included long axis [p = 0.01, OR = 3.06 (1.33–7.06)], S/L ratio [p = 0.047, OR = 2.15 (1.01–4.57)], prior history of malignancy [p = 0.01, OR = 2.97 (1.26–6.99)] and internal echogenicity [p = 0.01, OR = 2.72 (1.26–5.86)] (Table 3). We further performed a trend test to evaluate the consistency between the two variables (long axis, S/L ratio) and a final malignancy. The results showed significant positive correlations for these two variables (p < 0.05).

**Table 3:** Multivariate analyses performed by using a stepwise logistic regression model to identify the risk factors for malignant results in patients who had neck LNs with indeterminate cytology.

Variables	OR	95% CI	P value
Age	1.95	0.92–4.11	0.08
Sex	1.69	0.79–3.64	0.18
Short axis			
Long axis	3.06	1.33–7.06	**0.01** ^a)^
S/L ratio	2.15	1.01–4.57	**0.047** ^a)^
Prior history of malignancy	2.97	1.26–6.99	**0.01** ^a)^
Cytological classification	5.67	3.48–9.23	**< 0.001** ^a)^
Boundary			
Internal echogenicity	2.72	1.26–5.86	**0.01** ^a)^
Calcification			
Hilar echogenicity			
Vascular pattern			

Abbreviation: S/L, short-to-long axis; LNs, lymph nodes.

^a)^Statistical significance, p < 0.05.

We then used the cytological classification and other four variables (prior history of malignancy, long axis ≥ 1.93 cm, S/L ratio ≥ 0.64 and heterogeneity of internal echogenicity) in combination to weigh the malignancy rate. The final malignancy rate was 94% in the high-risk group, and we did not need to check the other four variables in the group. For the low- and moderate-risk groups, the malignancy rate increased with the number of positive significant variables (Table 4).

**Table 4:** The final malignancy rate was determined by using the cytological classification and other four predictors in patients who had neck LNs with indeterminate cytology.

Cytological classification	Four factors (prior history of malignancy, long axis ≥ 1.93 cm, S/L ratio ≥ 0.64, and heterogeneity of internal echo)	Malignancy rate
Low risk (mild atypia, focal atypical cells, or tumor necrosis)	All negative	2/18 (11%)
	1 positive	4/26 (15%)
	2 positive	5/16 (31%)
	3 positive	7/12 (58%)
	All positive	Nil
Moderate risk (atypia or some atypical cells)	All negative	0/12 (0%)
	1 positive	7/16 (44%)
	2 positive	11/24 (46%)
	3 positive	7/8 (88%)
	All positive	2/2 (100%)
High risk (highly atypia, highly atypical cells or suspicion of malignancy)		96/103 (93%)

Abbreviation: S/L, short-to-long axis; LNs, lymph nodes.

## Discussion

Occasionally, cytopathologists may not be able to report a unique diagnosis despite the sample being adequate for analysis, and this kind of result was deemed to be indeterminate. Indeterminate FNA cytology reportedly accounts for 8–10% of all cervical LN FNAs [3, 9]. In this series, the rate of indeterminate FNA cytology was 7%, which was similar to that in the previous reports. Furthermore, knowing the malignancy rate of indeterminate FNA cytology results can help us perform patient counseling as well as guide further management. Tarantino et al. [3] and Bandoh et al. [9] showed that the final malignancy rates of LNs with indeterminate cytology were 77% (10/13) and 79% (11/14), respectively. In our study, we analyzed 237 patients who had indeterminate cytological reports, and the final malignancy rate was 60%, which is slightly lower than that in the previous studies. The discrepancies among studies regarding the final malignancy rate in this population might be due to differences in the definition of indeterminate FNA cytology or interpretation among cytopathologists. Borrowing from the Bethesda classification for thyroid FNA diagnosis, the indeterminate group included atypia of undetermined significance, follicular neoplasm, and suspicion of malignancy [13]. Similarly, we proposed a cytological classification system for cervical LNs with indeterminate FNA cytology. We noted that the final malignancy rate was higher in reports noting a suspicion of malignancy or highly atypical cells and lower in those noting focal atypical cells or tumor necrosis. To the best of our knowledge, the current study is the first and largest study to further divide cervical LNs with indeterminate FNA cytology into 3 different risk groups.

Among the demographic data and sonographic features, age, sex, short axis, long axis, S/L ratio, prior history of malignancy, cytological classification, boundary, internal echogenicity, calcifications, hilar echogenicity and vascular pattern were related to a final malignant diagnosis (Table 2). Previous studies have shown that size, shape, margin, hilar echogenicity and vascular pattern are diagnostic factors for malignant neck LNs [10, 14]. The sonographer performed US-FNA of the suspicious LN with one or more of the abnormal features mentioned above. However, when the cytological reports reveal indeterminate results, no study has evaluated factors that predict if nodes are malignant or not in this circumstance. In this study, multivariate analyses with a stepwise logistic regression model adjusted by age and sex showed that cytological classification had the highest OR with a significant difference. Moreover, the other four variables (prior history of malignancy, long axis, S/L ratio and internal echogenicity) were also independent factors in predicting a final malignancy in patients who initially had nodal aspirations with indeterminate cytological results (Table 3). The size of the malignant node tends to become large as the tumor rapidly grows [11]. The shape of the malignant node tends to be round, and an increasing S/L ratio might be noted [11]. The internal echogenicity of the malignant node tends to be heterogeneous due to its necrotic and solid composition [15]. The neck recurrence rate in patients with a prior history of malignancy was not low [16, 17]; thus, these patients tend to have more malignant results. These four variables can be quickly evaluated during neck US and are easy to review with the reporting system after knowing the cytological reports.

In reality, unfortunately, head and neck surgeons are commonly forced to make clinical and operative decisions based on suboptimal conditions, such as indeterminate FNA results. According to the 2015 American Thyroid Association (ATA) guidelines for thyroid nodules with indeterminate cytological results, repeat FNA or molecular testing is recommended for the low malignancy risk group, diagnostic surgical excision is advised for the moderate risk group, and surgical management similar to that for malignant cytology results is suggested for those with a suspicion of malignancy, even though the estimated final malignancy rate is 60–75% [18]. Based on the ATA guidelines for thyroid nodules, we divided our patients according to the cytological classification system and the other four significant predictors in Table 4. The final malignancy rate was 94% in the high-risk group. For the low- and moderate-risk groups, the malignancy rate increased with more positive significant variables. As a result, for neck LAP with indeterminate cytological results, we can evaluate the cytological classification first and then check if there is a prior history of malignancy, long axis ≥ 1.93 cm, S/L ratio ≥ 0.64 and heterogeneity of internal echogenicity before making further decisions. For the high-risk group, we recommended directly managing the LAP as a malignancy. If no variables were positive in the low- and moderate-risk groups, the malignancy rates were 11% and 0%, respectively. Under these conditions, close observation or repeat FNA may be the appropriate treatment option. If one or two predictors were positive in the low- and moderate-risk groups, the malignancy rates were higher, and we suggested core needle biopsy or excisional biopsy to further confirm the diagnoses. If three or four variables were positive in the low- and moderate-risk groups, the malignancy rate was 58–100% in this series. We suggested handling the LAP as a malignancy in these circumstances.

### Limitations

There were several limitations in this study. First, not all our final diagnoses were based on the histopathologic findings. After a follow-up of at least 12 months, patients with nodes that diminished or were equal in size were deemed as negative. Inevitably, there may be a small chance that the node was positive when a slow-growing metastatic lesion, such as metastasis from papillary thyroid carcinoma, was encountered. Second, there could be bias in the results from including patients with a prior history of malignancy. We tried to divide the patients into a treatment-naïve group and a group with a prior history of malignancy. After the univariate and multivariate analyses, the cytological classification remained an independent factor (p < 0.001) with a high odds ratio in predicting a final malignancy in both groups (not reported in the present study). This meant that regardless of whether the patient was treatment naïve, the cytological classification could be used as the first priority to ascertain the malignancy risk in patients with indeterminate FNA results. In this series, we added one parameter of a prior history of malignancy to minimize the bias from heterogeneity by including treated and treatment-naïve patients. Third, the data were only center-based and lacked verification. There is no standard classification for defining mild atypical, atypical or highly atypical cells, and discrepancies might exist between the interpretations of cytopathologists. A standardized classification of the degree of the indeterminate FNA results will be greatly advantageous in the absence of a definitive diagnosis from FNA. Development of a standard classification system in the future can help determine patient risk for malignant cervical nodes and further guide clinical decision-making. Further large-scale and prospective studies are necessary in the future.

## Conclusion

Indeterminate FNA cytology in the evaluation of cervical LAP should raise the suspicion of malignancy. Cytological classification and four other predictors (prior history of malignancy, long axis ≥ 1.93 cm, S/L ratio ≥ 0.64 and heterogeneity of internal echogenicity) are all statistically associated with the risk of malignancy in this group of patients and are helpful in guiding further management.

## Supporting information

S1 Table Raw data set. (XLSX)

## References

[1] Chau I, Kelleher MT, Cunningham D, Norman AR, Wotherspoon A, Trott P, et al Rapid access multidisciplinary lymph node diagnostic clinic: analysis of 550 patients. Br J Cancer. 2003;88(3):354–61. Epub 2003/02/06. 10.1038/sj.bjc.6600738 . 12569376 PMC2747551

[2] Hafez NH, Tahoun NS. Reliability of fine needle aspiration cytology (FNAC) as a diagnostic tool in cases of cervical lymphadenopathy. J Egypt Natl Canc Inst. 2011;23(3):105–14. Epub 2012/07/11. 10.1016/j.jnci.2011.09.009 . 22776815

[3] Tarantino DR, McHenry CR, Strickland T, Khiyami A. The role of fine-needle aspiration biopsy and flow cytometry in the evaluation of persistent neck adenopathy. American journal of surgery. 1998;176(5):413–7. Epub 1999/01/05. 10.1016/s0002-9610(98)00233-5 . 9874424

[4] Johnson JT, Rosen CA, Bailey BJ. Bailey’s head and neck surgery otolaryngology 5th ed: Wolters Kluwer Health/Lippincott Williams & Wilkins, Philadelphia, PA; 2014 1760–87 p.

[5] Layfield LJ. Fine-needle aspiration in the diagnosis of head and neck lesions: a review and discussion of problems in differential diagnosis. Diagn Cytopathol. 2007;35(12):798–805. Epub 2007/11/17. 10.1002/dc.20769 . 18008348

[6] Borhani AA, Monaco SE. Chapter 7 Image-Guided Fine-Needle Aspiration and Core Needle Biopsy of Neck Lymph Nodes: Techniques, Pearls, and Pitfalls. Semin Ultrasound CT MR. 2017;38(5):531–41. Epub 2017/10/17. 10.1053/j.sult.2017.05.007 . 29031369

[7] Houcine Y, Romdhane E, Blel A, Ksentini M, Aloui R, Lahiani R, et al Evaluation of fine needle aspiration cytology in the diagnosis of cervical lymph node lymphomas. J Craniomaxillofac Surg. 2018;46(7):1117–20. Epub 2018/05/22. 10.1016/j.jcms.2018.04.024 . 29779620

[8] Goret CC, Goret NE, Ozdemir ZT, Ozkan EA, Dogan M, Yanik S, et al Diagnostic value of fine needle aspiration biopsy in non-thyroidal head and neck lesions: a retrospective study of 866 aspiration materials. International journal of clinical and experimental pathology. 2015;8(8):8709–16. Epub 2015/10/16. . 26464615 PMC4583847

[9] Bandoh N, Goto T, Akahane T, Ohnuki N, Yamaguchi T, Kamada H, et al Diagnostic value of liquid-based cytology with fine needle aspiration specimens for cervical lymphadenopathy. Diagn Cytopathol. 2016;44(3):169–76. Epub 2016/01/11. 10.1002/dc.23402 . 26748563 PMC5066749

[10] Liao LJ, Wang CT, Young YH, Cheng PW. Real-time and computerized sonographic scoring system for predicting malignant cervical lymphadenopathy. Head Neck. 2010;32(5):594–8. Epub 2009/08/21. 10.1002/hed.21225 . 19693943

[11] Ahuja AT, Ying M, Ho SY, Antonio G, Lee YP, King AD, et al Ultrasound of malignant cervical lymph nodes. Cancer Imaging. 2008;8:48–56. Epub 2008/04/09. 10.1102/1470-7330.2008.0006 . 18390388 PMC2324368

[12] Pusztaszeri MP, Faquin WC. Cytologic evaluation of cervical lymph node metastases from cancers of unknown primary origin. Semin Diagn Pathol. 2015;32(1):32–41. Epub 2015/01/27. 10.1053/j.semdp.2014.12.002 . 25618223

[13] Cibas ES, Ali SZ. The 2017 Bethesda System for Reporting Thyroid Cytopathology. Thyroid: official journal of the American Thyroid Association. 2017;27(11):1341–6. Epub 2017/11/02. 10.1089/thy.2017.0500 . 29091573

[14] Rotim T, Kristek B, Turk T, Kretic D, Peric M, Puseljic I, et al Measurable and Unmeasurable Features of Ultrasound Lymph Node Images in Detection of Malignant Infiltration. Acta clinica Croatica. 2017;56(3):415–24. Epub 2018/02/27. 10.20471/acc.2017.56.03.08 . 29479907

[15] Yoshida H, Yusa H, Ueno E, Tohno E, Tsunoda-Shimizu H. Ultrasonographic evaluation of small cervical lymph nodes in head and neck cancer. Ultrasound in medicine & biology. 1998;24(5):621–9. Epub 1998/08/08. 10.1016/s0301-5629(98)00025-8 . 9695264

[16] Chang JH, Wu CC, Yuan KS, Wu ATH, Wu SY. Locoregionally recurrent head and neck squamous cell carcinoma: incidence, survival, prognostic factors, and treatment outcomes. Oncotarget. 2017;8(33):55600–12. Epub 2017/09/15. 10.18632/oncotarget.16340 . 28903447 PMC5589686

[17] Amar A, Chedid HM, Rapoport A, Dedivitis RA, Cernea CR, Brandao LG, et al Update of assessment of survival in head and neck cancer after regional recurrence. J Oncol. 2012;2012:154303 Epub 2012/11/06. 10.1155/2012/154303 . 23125856 PMC3483779

[18] Haugen BR, Alexander EK, Bible KC, Doherty GM, Mandel SJ, Nikiforov YE, et al 2015 American Thyroid Association Management Guidelines for Adult Patients with Thyroid Nodules and Differentiated Thyroid Cancer: The American Thyroid Association Guidelines Task Force on Thyroid Nodules and Differentiated Thyroid Cancer. Thyroid: official journal of the American Thyroid Association. 2016;26(1):1–133. Epub 2015/10/16. 10.1089/thy.2015.0020 . 26462967 PMC4739132

